# Real-world experiences of CNS-directed chemotherapy followed by autologous stem cell transplantation for secondary CNS involvement in relapsed or refractory diffuse large B-cell lymphoma

**DOI:** 10.3389/fonc.2022.1071281

**Published:** 2023-01-19

**Authors:** Sun Young Jeong, Sang Eun Yoon, Duck Cho, Eun Suk Kang, Junhun Cho, Won Seog Kim, Seok Jin Kim

**Affiliations:** ^1^ Division of Hematology-Oncology, Department of Medicine, Samsung Medical Center, Sungkyunkwan University School of Medicine, Seoul, Republic of Korea; ^2^ Department of Laboratory Medicine, Samsung Medical Center, Sungkyunkwan University School of Medicine, Seoul, Republic of Korea; ^3^ Department of Pathology, Samsung Medical Center, Sungkyunkwan University School of Medicine, Seoul, Republic of Korea; ^4^ Department of Health Sciences and Technology, Samsung Advanced Institute for Health Sciences and Technology, Sungkyunkwan University, Seoul, Republic of Korea

**Keywords:** secondary CNS involvement, autologous stem cell transplantation, diffuse large B-cell lymphoma, salvage treatment, high-dose methotrexate (HD-MTX)

## Abstract

**Introduction:**

Secondary central nervous system (CNS) involvement is a rare but fatal event in patients with diffuse large B cell lymphoma (DLBCL). Some studies have suggested autologous stem cell transplantation (ASCT) for patients responding to salvage therapies, although its role is not clear.

**Methods:**

We analyzed DLBCL patients with secondary CNS involvement who received salvage therapies with curative intent and who underwent high-dose chemotherapy followed by ASCT. We analyzed the post-ASCT outcome in terms of CNS and/or systemic relapse and overall survival (OS) according to type of secondary CNS involvement and salvage treatment.

**Results:**

A total of 43 patients who achieved complete or partial response after salvage treatments, mainly high-dose methotrexate (MTX)-containing chemotherapy, was treated with busulphan-thiotepa followed by ASCT between 2009 to 2019. Fifteen patients experienced grade III/IV febrile neutropenia, but all adverse events were manageable. At the median follow-up of 14.7 months after ASCT, 17 patients did not relapse, however, 26 patients had relapsed, comprising isolated CNS relapse (n = 12), systemic relapse (n = 12), and both (n = 2). Patients with systemic relapse had significantly shorter OS than those with isolated CNS relapse (42.7 vs, 11.1 months, p = 0.002). Of the 26 patients who relapsed after ASCT, six patients were rescued by subsequent salvage treatments. Finally, 21 patients were alive at the time of analysis.

**Discussion:**

In conclusion, consolidative ASCT might be beneficial for secondary CNS involvement in relapsed or refractory DLBCL patients if they responded to CNS-directed salvage chemotherapy and were eligible for transplantation.

## Introduction

Secondary central nervous system (CNS) involvement is an uncommon complication of diffuse large B cell lymphoma (DLBCL) with around 5% incidence, but it carries a poor prognosis (1, 2). Secondary CNS involvement can be observed as a sign of disease progression during treatment for systemic DLBCL or as a relapse disease with or without systemic relapse of DLBCL. The clinical outcome of DLBCL patients with secondary CNS involvement is dismal because the CNS is a chemotherapy sanctuary site and secondary CNS involvement demonstrates the aggressiveness of systemic DLBCL (3–5). Although there is no consensus on optimal treatment strategies for secondary CNS involvement, treatments targeting CNS lesions have been widely used, such as treatment for primary CNS lymphoma. Thus, high-dose methotrexate (MTX)-containing chemotherapies might be effective for patients with secondary CNS involvement. However, the majority of patients fail to be cured by these therapeutic approaches because the response duration is relatively short and systemic disease progression can occur outside the CNS (6, 7).

Nevertheless, CNS-directed high-dose chemotherapy followed by autologous stem cell transplantation (ASCT) has been conducted for transplant-eligible patients because previous phase II studies demonstrated ASCT as a feasible consolidative treatment to improve the survival outcome of secondary CNS involvement (8–12). However, due to the rarity of the disease, those studies have small and heterogeneous populations with different histologies, first-line therapies, salvage therapies, and conditioning regimens. Furthermore, a substantial number of patients with secondary CNS involvement might not be enrolled in prospective studies because they are usually in poor health condition. Therefore, real-world data representing the efficacy and feasibility of ASCT in clinical practice are needed. Previous retrospective studies have shown a prolonged duration of response in a small number of patients who received high-dose busulfan/thiotepa-based conditioning and ASCT (13, 14). In addition, an international multicenter retrospective study analyzing 291 DLBCL patients with secondary CNS involvement also reported favorable outcome of patients who underwent ASCT after intensive salvage chemotherapy with curative intent (15). However, only 25 of those patients received ASCT after heterogeneous conditioning regimens. Thus, there are limited data regarding the efficacy of ASCT in DLBCL patients with secondary CNS involvement. This single-center real-world data study analyzed the duration of response and survival outcome of DLBCL patients with secondary CNS involvement who underwent ASCT as a consolidation treatment and compared the outcome after ASCT based on the pattern of secondary CNS involvement.

## Methods

### Patients

We retrospectively reviewed the medical records of patients who were diagnosed with DLBCL from 2009 to 2019, and selected patients according to the following criteria: 1) CNS relapse or progression during or after R-CHOP chemotherapy; 2) underwent ASCT after salvage treatments for secondary CNS involvement; and 3) data available for analysis of disease relapse and survival status. We excluded patients who had CNS involvement at diagnosis. As this study aimed to evaluate the role of ASCT in DLBCL patients with secondary CNS involvement, patients who did not undergo ASCT were also excluded. Patient demographics and clinical characteristics at initial presentation and at the time of secondary CNS involvement were collected, and information about the type of salvage therapy after secondary CNS involvement and ASCT was gathered. For patients who relapsed after ASCT, the patterns of relapse and post-relapse outcomes were analyzed, and survival status was last updated in April 2022. The Institutional Review Board of Samsung Medical Center approved this study (IRB No: 2022-05-035), and informed consent was waived due to the retrospective nature.

### Diagnosis of secondary CNS involvement

The evaluation of CNS was conducted for patients having signs and/or symptoms of suspicious CNS involvement during or after R-CHOP chemotherapy. The diagnosis of CNS involvement was based on the combination of neurologic manifestations, magnetic resonance imaging (MRI), and/or histological findings of the brain parenchyma or cerebrospinal fluid (CSF). The patterns of CNS involvement were parenchymal, leptomeningeal, and combined involvement. Parenchymal involvement was diagnosed on brain MRI or brain biopsy. If the cytologic examination of CSF showed the presence of lymphoma cells or suspicious lymphoma cells with increased protein levels, leptomeningeal involvement was diagnosed. In addition, when leptomeningeal enhancement compatible with leptomeningeal involvement was observed in brain or spine MRI of patients with neurologic symptoms or signs, leptomeningeal involvement was diagnosed.

### ASCT and response evaluation

ASCT was carried out for patients who responded to salvage chemotherapy after CNS involvement. Peripheral blood stem cells were collected during salvage chemotherapy or mobilization chemotherapy with etoposide as we previously reported for patients with primary CNS lymphoma (16). The target number of CD34+ cells was more than 3.0 × 10^6^ per kilogram of the recipient’s body weight. The conditioning regimen performed prior to ASCT was the same as that for primary CNS lymphoma, consisting of busulfan and thiotepa (busulfan 3.2 mg/kg days –9 to –5, and thiotepa 5 mg/kg days –4 to –3) as we previously reported (16, 17). Treatment response was assessed according to the involved sites. CNS response evaluation was assessed by contrast-enhanced MRI with neuroradiologic review according to the international guidelines for primary CNS lymphoma (18). If a patient had simultaneous systemic disease involvement, systemic response evaluation was also performed according to the Lugano response criteria (19).

### Statistical analysis

The clinical features and treatment outcomes were analyzed, and categorical variables were evaluated using Fisher’s exact test. The time to secondary CNS involvement was defined as the time between the initial diagnosis of DLBCL to the date of secondary CNS involvement. The post-ASCT overall survival (OS) was defined as the time from the date of stem cell infusion to death from any cause, and living patients were censored at the time of analysis. The post-ASCT progression-free survival (PFS) was defined as the time from the date of stem cell infusion to the date of systemic or CNS progression or relapse or death. Survival was estimated based on Kaplan–Meier curves and compared using a log-rank test. Two-sided statistical tests yielding a P value < 0.05 were considered significant. Survival analyses were performed using IBM PASW version 24.0 software (SPSS Inc., Chicago, IL, USA).

## Results

### Patients with secondary CNS involvement

**Figure 1:**
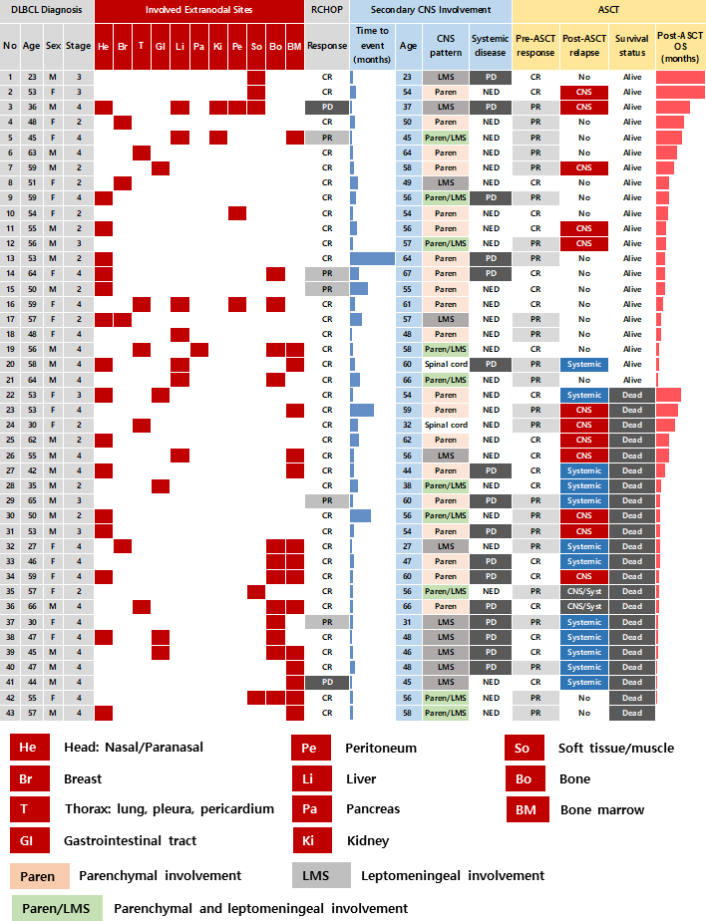
Summary of the 43 patients in the study.

**Figure 2:**
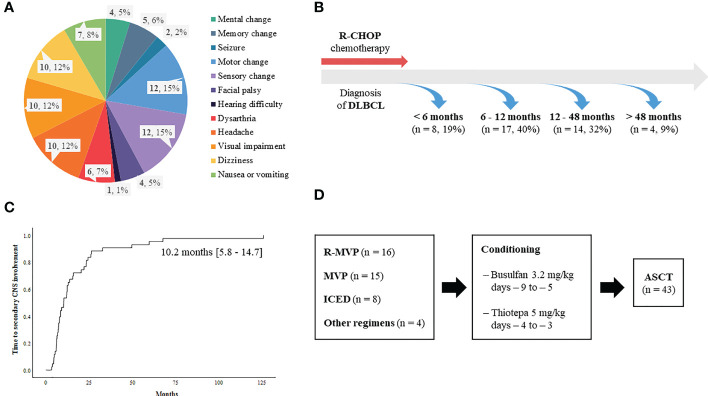
Characteristics of secondary CNS involvement in relapsed or refractory DLBCL patients **(A)** Signs and symptoms suspicious of secondary CNS involvement leading to CNS evaluation **(B)** Onset of secondary CNS involvement **(C)** Time to secondary CNS involvement **(D)** Treatment summary of 43 patients.

A total of 92 patients had secondary CNS involvement at our institute between 2009 and 2019 according to the review of medical records. As the ASCT was only reimbursed for patients who were younger than 65 years old during that period, 70 patients were determined to be eligible for ASCT based on the reimbursement criteria. However, out of 70 patients who were potentially eligible for ASCT, 43 patients (43/70, 61.4%) underwent ASCT after they achieved complete or partial response to CNS-directed salvage chemotherapy and the remaining patients (n = 27) could not receive ASCT because they failed to respond to salvage therapy. Thus, we analyzed 43 patients who underwent ASCT after secondary CNS involvement. Their median age at initial diagnosis of DLBCL was 53 years (range: 23 – 66 years), and 23 patients were male (54%). The majority of patients had ECOG 0/1 (n = 38, 88%) at diagnosis, and initial stages were as follows: stage II (n = 13, 30%), III (n = 6, 14%), and IV (n = 24, 56%). However, extranodal involvement was found in most patients, and bone marrow was involved in 15 patients (35%) at diagnosis. The presence of at least one extranodal involvement was found in 43 patients (95%), and 19 patients (44%) had two or more extranodal involvements (**Figure 1**). The involvement of a head area adjacent to the brain such as nasal cavity and paranasal sinuses was most common (n = 16) compared to other organs including liver (n = 7), gastrointestinal tract (n = 5), thorax (n = 5), soft tissue/muscle (n = 5), breast (n = 4), and kidney (n = 2). However, out of 43 patients who underwent ASCT, there was no case of DLBCL that initially had testicular involvement at diagnosis. All patients received R-CHOP as the primary treatment, and the response was as follows: CR (n = 36, 84%), PR (n = 5, 12%), and PD (n = 2, 5%). During R-CHOP chemotherapy, only eight patients received CNS prophylaxis according to physicians’ discretion in case of breast involvement or high tumor burden. Thus, six patients received IT MTX (intrathecal administration of 12 mg of MTX every three weeks) whereas two patients received high-dose MTX prophylaxis (MTX 3.5g/m^2^ intravenous infusion every three weeks for two cycles after the completion of R-CHOP chemotherapy). However, CNS involvement occurred even in patients with stage II completely responding to R-CHOP (Patient No. 8, 25, 35, **Figure 1**). Secondary CNS involvement occurred as an isolated CNS relapse or progression in 27 patients (63%), whereas 16 patients (37%) showed concomitant systemic disease progression (**Figure 1**). The diagnosis of secondary CNS involvement was based on various manifestations suspicious of CNS involvement, and most patients presented multiple neurologic symptoms (**Figure 2A**). Secondary CNS involvement occurred most commonly within 12 months after initial diagnosis of DLBCL (**Figure 2B**); thus, the median age at the time of secondary CNS involvement was 55.7 years (range: 23.4 – 66.8 years) as the median time to secondary CNS involvement was 10.2 months (95% CI: 5.8 – 14.7 months, **Figure 2C**). At diagnosis of CNS involvement, 40% of patients had poor performance status (≥ ECOG 2), and the primarily involved sites of CNS were brain parenchyma (n = 20), leptomeninges (n = 11), both parenchyma and leptomeninges (n = 10), and spinal cord only (n =2). Patients received CNS-directed chemotherapy, mainly high-dose MTX-containing regimens as a salvage regimen. The salvage treatments that patients received after secondary CNS involvement were R-MVP (rituximab, methotrexate, vincristine, and procarbazine, n = 16), MVP (methotrexate, vincristine, and procarbazine, n = 15), ICED (ifosfamide, carboplatin, etoposide, and dexamethasone, n = 8), and other MTX-containing regimens (**Figure 2D**). The R-MPV treatment protocol consisted of rituximab (375mg/m^2^ intravenous on day 1), MTX (3.5g/m^2^ intravenous over 6 hours on day 2), leucovorin (15mg/m^2^ intravenous four times per day, total 12 times during three days), procarbazine (100mg/m^2^ orally administered for seven days at odd-numbered chemotherapy sessions), and vincristine (1.4mg/m^2^ intravenous on day 2) for a total of five cycles at two-week intervals. Among patients who received high-dose MTX, 28 received the planned dosage (3.5 g/m^2^), while three received less than 3.5 g/m^2^. The ICED treatment protocol consisted of intravenous ifosfamide 1670 mg/m^2^ on days 1-3, carboplatin 5 x AUC on day 1, intravenous etoposide 100 mg/m^2^ on days 1-3, and oral or intravenous dexamethasone 40mg on days 1–4 every three weeks, for four cycles.

### Outcome of ASCT

**Figure 3:**
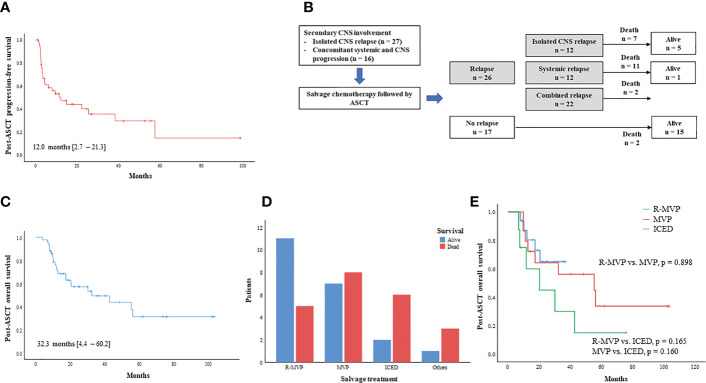
Outcome of ASCT for secondary CNS involvement **(A)** Progression-free survival after ASCT **(B)** Outcome after ASCT and type of post-ASCT relapse **(C)** Overall survival after ASCT **(D)** Comparison of survivors according to type of salvage CNS-directed treatment **(E)** Post-ASCT overall survival based on type of CNS-directed chemotherapy.

The median number of infused CD34+ cells per body weight was 4.1 × 10^6^ cells per kg (range: 2.0 – 34.9 × 10^6^). Fifteen patients experienced grade III/IV febrile neutropenia including one patient requiring treatment in the intensive care unit. Sinus obstruction syndrome occurred in one patient, however, all those adverse events were manageable. Neutrophil engraftment (the first of three days on which the neutrophil count was maintained at 500 × 10^6^/L) and platelet engraftment (the first day free from platelet transfusion following at least seven days with a platelet count >20 × 10^9^/L) occurred around nine days after ASCT. At the median follow-up of 14.7 months (95% CI: 3.7 – 25.7) after ASCT, 17 patients did not relapse. However, the remaining 26 patients (60.5%) relapsed, and more than 60% of relapses were found (n = 16, 61.5%) within six months after ASCT. Thus, the median PFS after ASCT was 12.0 months (95% CI: 2.7 – 21.3 months, **Figure 3A**). The patterns of relapse were isolated CNS, systemic, and combined relapses, and 20 patients died after relapse. Out of 26 relapsed patients, six patients were alive after subsequent salvage treatments (**Figure 3B**). Thus, the median OS after ASCT was 32.3 months (95% CI: 4.4 – 60.2, **Figure 3C**). The number of survivors was significantly higher in patients receiving R-MVP after secondary CNS involvement (**Figure 3D**). Thus, the OS of patients receiving R-MVP or MVP was better than that of those receiving ICED after secondary CNS involvement, although the difference was not statistically significant (**Figure 3E**). The IT MTX was administered simultaneously to 15 patients including 11 patients with leptomeningeal involvement, however, the use of IT MTX for patients with leptomeningeal involvement did not have any impact on their survival outcome. Thus, the OS of patients who received IT MTX plus R-MVP or MVP was not significantly different from that of patients who received R-MVP or MVP alone (p = 0.255, data not shown). In case of isolated CNS relapse, R-MVP or MVP were mainly used as salvage treatments without whole-brain radiotherapy. Radiotherapy including whole-brain radiotherapy or gamma-knife was done in 13 patients, especially for concomitant systemic and CNS progression and patients who were symptomatic for their brain lesion.

### Post-ASCT relapse

**Figure 4:**
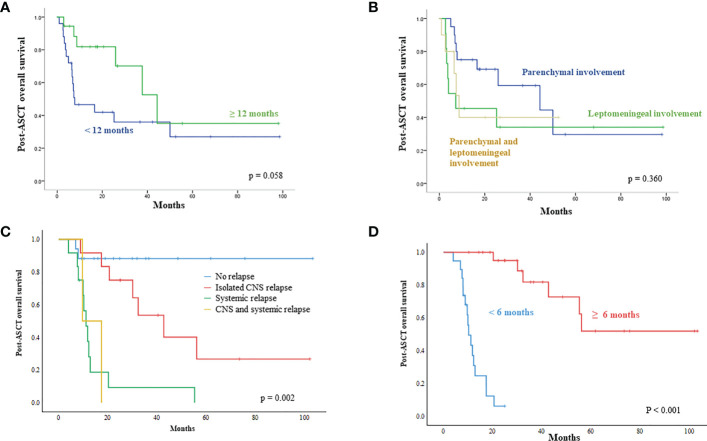
Unfavorable parameters influencing survival outcome after ASCT **(A)** Patients demonstrating secondary CNS involvement within 12 months after initial diagnosis showed worse survival after ASCT **(B)** Patients having leptomeningeal involvement show a tendency for poor survival after ASCT **(C)** Systemic relapse after ASCT has a negative impact on post-ASCT survival **(D)** Early relapse within six months after ASCT showed worse overall survival than relapse occurring after six months.

The post-ASCT OS was compared by the time of onset of secondary CNS involvement after initial diagnosis. Patients with early secondary CNS involvement within 12 months showed worse OS than patients with secondary CNS involvement 12 months later, although the difference was not significant (**Figure 4A**). The comparison of survival outcomes according to the type of CNS involvement showed that the presence of leptomeningeal involvement had a negative effect on survival, although it was not statistically significant (**Figure 4B**). In addition, patients who experienced an isolated CNS relapse after ASCT showed significantly better OS than patients with systemic or combined relapse (**Figure 4C**). The onset of relapse after ASCT was also related to prognosis after ASCT, thus, post-ASCT relapse within six months after ASCT showed poor OS, and the majority of those patients with early relapse experienced systemic relapse (**Figures 1**, **4D**).

## Discussion

Our study evaluated the outcomes of 43 DLBCL patients with secondary CNS involvement who underwent successful ASCT after salvage chemotherapy with curative intent. As secondary CNS involvement is not a common event in DLBCL, and only patients responding to salvage chemotherapy could undergo ASCT, the proportion of patients receiving ASCT is small even among patients with secondary CNS involvement (13–15). Indeed, the feasibility of ASCT is limited due to the failure of salvage treatment, toxicity, and unsuccessful stem cell harvest. Thus, ASCT has been reported as being applicable only to selected patients with secondary CNS involvement (20). Therefore, this study analyzed a relatively large number of patient outcomes according to onset and type of secondary CNS involvement.

Secondary CNS involvement could occur as isolated CNS relapse or progression or concomitant with systemic progression. The previous study that gathered the largest number of DLBCL patients with secondary CNS involvement showed a median time from initial diagnosis of DLBCL to secondary CNS involvement of nine months (range: 1 – 132 months) (15). A previous multicenter prospective study on secondary CNS involvement reported a median time of 10.4 months (range: 3.4 – 29.2 months) from diagnosis to secondary CNS involvement (21). Our study likewise showed 10.2 months of median time to CNS involvement, and around 60% of cases occurred within 12 months after the initial diagnosis of DLBCL. Although it was not statistically significant, OS after ASCT was inferior when secondary CNS involvement occurred within 12 months of initial diagnosis compared to when CNS relapse occurred after 12 months. The pattern of CNS involvement was also associated with the prognosis of secondary CNS involvement. Brain parenchyma is the most commonly reported site with a better outcome than leptomeninges (15). Our study also showed the negative impact of leptomeningeal involvement on the outcome of patients after ASCT, although it was not significant.

As high-dose MTX is the mainstay of CNS-directed treatment, the majority of patients (83.7%) in our study received high-dose MTX-containing chemotherapy with curative intent. Especially, 16 patients received treatment in combination with rituximab, such as R-MVP, because R-MVP followed by ASCT has been widely used for the treatment of primary CNS DLBCL (16, 22). The outcome after ASCT was better in patients receiving R-MVP or MVP than in patients receiving ICED. Likewise, a retrospective study of 113 patients with isolated CNS relapse of systemic NHL reported better OS in the MTX group (p=0.007) (23). Our study showed that 15 patients maintained their response without evidence of relapse, demonstrating a plateau in the survival curve. Nevertheless, 26 patients experienced relapse after ASCT, and relapses occurred in the CNS, systemically, or both. In particular, patients who had systemic relapse showed significantly shorter OS than those who had isolated CNS relapse. The median time to relapse after ASCT was 12.0 months (range; 2.1-21.3 months). The patients that relapsed within six months after ASCT tended to have a systemic relapse; only four of them had isolated CNS relapse (**Figure 1**). This might be why early relapsed patients had shorter OS. Our survival outcome after ASCT was comparable to that of previous prospective studies analyzing patients with secondary CNS involvement (**Table 1**). Among previous studies, one study reported 32% OS at one year, after the use of busulfan/cyclophosphamide as a conditioning regimen (11). Considering that the thiotepa-based conditioning regimen was associated with longer OS in primary CNS lymphoma (24), the type of conditioning could influence the post-ASCT outcome.

**Table 1:** Summary of previous studies.

	Ferreri et al. (12)	Doorduijn et al. (11)	Ferreri et al. (10)	Korfel et al (8)	Our study
No. of patients	75	36	38	30	92
Median age (range)	58 (23-70)	57 (23-65)	59 (36-70)	58 (29-65)	51 (23-66)
Median time to CNS involvement (month, range)	Not reported	12 (2-186)	1 (0-69)	9 (3-80)	10 (3-126)
CNS involvement at relapse (%)	57	100	58	100	100
Transplant patients (%)	49	42	53	80	46
PFS after ASCT	1y 58%	1y 19%	2y 50%	2y 49%	2y 58.6%
Median OS after CNS relapse (month)	29	7	Not reported	Not reported	32
OS of transplant patients	2y 83%	1y 32%	2y 68%	2y 68%	2y 57%
Conditioning regimen	Carmustine Thiotepa	Busulfan Cyclophosphamide	BCNU Thiotepa	Carmustin Thiotepa Etoposide	Busulfan Thiotepa

Although our results showed that high-dose MTX-based salvage therapy and ASCT might be beneficial for patients with secondary CNS involvement if they qualify for transplantation, our findings could be influenced by selection bias because we only analyzed patients receiving ASCT who might have a better prognosis than those who did not receive ASCT. Thus, the role of ASCT in DLBCL patients with secondary CNS involvement in this study should be cautiously interpreted given the limitation of our study. However, active application of intensified treatment followed by ASCT should be considered for patients who are eligible for transplantation, especially for cases with isolated CNS relapse 12 months after initial DLBCL diagnosis. However, considering that the prognosis of patients with relapse within six months after ASCT and those with concomitant systemic relapse was extremely poor, careful selection of those who may benefit from more intensive therapy is important, and additional therapeutic approaches should be explored in the future.

## Data availability statement

The raw data supporting the conclusions of this article will be made available by the authors, without undue reservation.

## Ethics statement

The study was approved by the Institutional Review Board of Samsung Medical Center (IRB number 2022-05-035).

## Author contributions

SJ and SK wrote the manuscript. DC and EK performed targeted sequencing and analysis. SJ and SK analyzed the data. SY, WK, and SK reviewed the clinical data. JC reviewed the pathology, and SK designed the study. All authors contributed to the article and approved the submitted version.
